# Assessment of Knowledge of Communicable Diseases Among Medical Students at Al-Balqa Applied University

**DOI:** 10.7759/cureus.55572

**Published:** 2024-03-05

**Authors:** Mais Alkhalili, Osama Bani Hani, Yamamah Al-Hmaid, Anees Hjazeen, Mohammad Hattab, Zeina Khraisat, Wala' AlDmour, Nanci Abdelrahim, Heba Abu Lubad

**Affiliations:** 1 Department of Public Health and Community Medicine, Al-Balqa Applied University, Al-Salt, JOR; 2 Department of General Surgery, Jordan University of Science and Technology, Irbid, JOR; 3 Department of Biostatistics, Jordanian Royal Medical Services, Amman, JOR; 4 Department of Community Medicine, Ministry of Health Holdings, Amman, JOR

**Keywords:** university, medical students, cholera, measles, tuberculosis, hepatitis b, leishmaniasis, communicable diseases, knowledge

## Abstract

Introduction: Medical education is the foundation of knowledge among medical students. This study aims to investigate the knowledge of medical students at Al-Balqa Applied University, exploring their awareness of five communicable diseases, namely, leishmaniasis, hepatitis B, tuberculosis, measles, and cholera.

Methods: This cross-sectional survey included 271 participants who answered a structured validated questionnaire with varying questions on causes, symptoms, complications, transmission routes, and preventive measures for each disease.

Results: Knowledge of all five communicable diseases was low. Leishmaniasis knowledge was notably low (mean=6.07, SD=1.43), with participants grappling with misconceptions about transmission modes, symptoms, and preventability. Hepatitis B knowledge was also low (mean=10.46, SD=1.67), especially regarding transmission modes, revealing that 76% of students were unaware of how the virus spreads. Tuberculosis knowledge unveiled gaps (mean=7.007, SD=1.90), particularly in recognizing the causes, symptoms, and transmission routes. Measles knowledge (mean=9.56, SD=1.92) indicated a robust understanding of symptoms but unveiled misconceptions about complications and transmission routes. For cholera (mean=14.50, SD=1.98), a knowledge of symptoms was demonstrated, but confusion about causative agents, transmission routes, and preventive measures was highlighted.

Conclusion: The findings of the study emphasize the critical need for enhanced educational strategies including curriculum revisions, increased practical exposure, engaging awareness campaigns, and the integration of interactive learning methods to increase knowledge about communicable diseases.

## Introduction

Communicable diseases are defined as diseases that can be spread from person to person or from animal to human [1]. These diseases can be classified into different categories according to symptoms, the route of transmission, and prevention. They are also classified according to the causative organism into viral, bacterial, fungal, and parasitic. Some communicable diseases, referred to as notifiable, should be reported to health authorities, such as cholera, measles, severe acute respiratory syndrome (SARS), and avian flu [2]. Communicable disease continues to be a major public health threat, due to the diversity of microorganisms causing it and the ability of these microorganisms to adapt and mutate into more aggressive types. The burden of communicable diseases continues to emerge worldwide as a major cause of mortality, especially in low-income countries and marginalized communities. Examples include human immunodeficiency virus (HIV), tuberculosis (TB), hepatitis, and neglected tropical diseases [3,4].

The fact that microbial resistance is emerging dramatically increases the risk of communicable diseases, even more than before. For instance, millions of people still pass away from TB each year, despite the availability of TB drugs and the possibility of treating multidrug-resistant TB [5]. ‏

The neglected tropical illness known as cutaneous leishmaniasis is spread throughout the world by female sandflies. Cutaneous leishmaniasis is endemic in Jordan, where the first case was documented in 1929. Treatment for cutaneous leishmaniasis is free in Jordan. There is no national control program, and the only form of surveillance is passive case detection with weekly reporting from local health department doctors [6].

With more than 10 million infected and 1.4 million deaths from TB in 2019, TB is regarded as one of the major global health threats and the second cause of death after coronavirus disease 2019 (COVID-19) [7]. More than half a million people infected with TB develop resistance each year. These statistics pose an enormous human and societal toll on a disease that could be prevented and cured, and they increase the need for measurements to raise knowledge and awareness about TB [8].

Regarding measles, outbreaks typically occur in late winter and early spring every year in temperate climates and are related to schools and overcrowded areas. Due to national vaccination programs, the incidence of measles has largely decreased, especially among the young. The age distribution of measles has shifted into adolescence and adulthood due to increased vaccination programs in the young. In addition to outbreaks in areas which have decreased vaccination rates, outbreaks of measles also occur in vulnerable populations such as the immunocompromised [9]. It is worth mentioning that Jordan had an outbreak of measles in 2023, mostly due to compromised immunity and vaccination programs following the COVID-19 pandemic [10].

It was estimated that around 60 million people are infected with hepatitis B in the Mediterranean region. In Jordan, the national prevalence of hepatitis B was around 2.4% in 2016. Studies in Jordan also reported an unexplained increase in the prevalence of hepatitis B-infected blood donations in 2019 and in pregnant women at around 5%. Vaccination is the main prevention strategy for hepatitis B and was adopted by the Jordanian Ministry of Health since 1995 for all newborns [11].

Annual reports concerning cholera had showed that around four million people are infected each year, with 143 deaths. This number is huge enough to consider cholera a public health threat [12]. Cholera is mainly distributed through contaminated food and water, and many areas of the world still encounter outbreaks of this bacteria. The poor knowledge about how cholera is transmitted is among the main contributors to the spread of the disease [13].

Medical students as a major part of health delivery systems are at risk as other healthcare workers when they come in contact with patients and contaminated instruments. They are expected to undertake activities related to patient care at the beginning of their clinical years [14].

To date, very few studies have been conducted to find out the knowledge of medical students about communicable diseases in Jordan and even worldwide. There should exist the ability to create a more coherent and powerful message about communicable diseases among medical students by including this subject in the medical curriculum or directing continuing actions in training, research, and publication.

This study aims to report the knowledge about five communicable diseases among medical students at Al-Balqa Applied University including cutaneous leishmaniasis, measles, hepatitis B, TB, and cholera.

## Materials and methods

Methodology

An observational cross-sectional design was adapted to achieve the study aim; a non-probability convenience sampling technique was used to collect the data from the medical students at Al-Balqa Applied University in Al-Salt, Jordan. The inclusion criteria were fourth- and fifth-year medical students who are registered at Al-Balqa Applied University. This deliberate selection criterion aimed to ensure that the participants possessed a certain level of academic progression, as fourth- and fifth-year students typically advance beyond basic medical courses, acquiring more in-depth knowledge and expertise in the field. By focusing on this cohort, the study aimed to capture insights from individuals who had progressed beyond introductory courses and were likely to possess a more comprehensive grasp of the subject matter under investigation.

This approach also facilitated the establishment of a homogenous sample, reducing potential confounding variables and enhancing the internal validity of the study. The utilization of a convenient sample from a specific academic level at Al-Balqa Applied University allowed for a targeted exploration of the research objectives, providing valuable insights into the knowledge levels of medical students who had already undergone a substantial portion of their medical education.

Sample size calculation

To ensure the adequacy of the sample, the sample size was calculated based on Slovin's formula, n=N/(1+Ne^2^), where N is the population size, which is in our case 694, and e is the error at 95% confidence interval, which will be 0.05, resulting in a calculated sample size of 254, which was considered a representative sample.

Study tool

The study tool utilized in this research project underwent a meticulous development process, drawing inspiration from a comprehensive review of existing literature [15-20]. To enhance its reliability and content validity, rigorous verification and cross-checking procedures were implemented. Experts in the relevant field were actively involved in the tool development, ensuring that the questions were not only clear but also aligned with the objectives of the study.

To further strengthen the study instrument's robustness, adjustments were made based on valuable suggestions and guidance received from the experts. This iterative process aimed at refining the questionnaire, aligning it more closely with the research objectives, and ensuring its overall efficacy.

The final version of the validated questionnaire comprised 28 items, structured in a "Yes," "No," and "I don't know" format. These items were strategically categorized into distinct sections, encompassing 14 items on leishmaniasis, 21 on hepatitis b, 18 on TB, 20 on measles, and 29 on cholera. This systematic organization allowed for a comprehensive exploration of participants' knowledge across various infectious diseases.

To assess the questionnaire's practicality and effectiveness, a pilot study involving 25 medical students was conducted. This preliminary evaluation served as a crucial step in identifying potential issues, gauging the clarity of the questions, and refining the tool for optimal use in the main study. Adjustments and fine-tuning were undertaken based on the insights gained from the pilot study, ultimately contributing to the robustness and reliability of the research tool.

The final version of the questionnaire was distributed to the students through Google Forms, after fully explaining the study and its objectives and obtaining the consent which was signed as a part of the online questionnaire.

Data analysis

Descriptive statistics including means and frequencies were used to report the study results. A score of 1 was given for each correct answer and 0 for an incorrect response. Based on the total score, the level of knowledge was classified into "low-level knowledge," "moderate-level knowledge," and "high-level knowledge." The score of each level was computed based on the number of questions specific to each disease. Cumulative scores were used to classify the participants into distinct levels of knowledge. The categorization included three tiers: "low-level knowledge," "moderate-level knowledge," and "high-level knowledge." The determination of knowledge levels was intricately tied to the total score achieved by each participant, which was computed based on the number of correct answers specific to each disease category.

Reliability analysis

The Kuder-Richardson 21 was used to measure the internal consistency of the study tool due to the binary format of answers. The result in Table 1 shows that the reliability coefficients ranged from 0.755 to 0.825 which is above the threshold cutoff point 0.70 indicating that the study tool is reliable to use for data collection.

**Table 1:** The reliability coefficients of the five sections

Disease section	Number of items	Kuder-Richardson coefficients
Leishmaniasis transmission	14	.772
Hepatitis B	21	.825
Tuberculosis disease	18	.810
Measles	20	.755
Cholera	29	.799

Ethical considerations

Approval was obtained from the Institutional Review Board of Al-Balqa Applied University (approval number: 2024/2023/3/25). Data was treated with confidentiality, and the privacy of the participants was maintained. Participation was voluntary, and participants had the right to withdraw at any time during the study conduction.

## Results

Leishmaniasis transmission

The participants answered 14 questions that examined their knowledge about leishmaniasis. Generally, a noticeable proportion of participants incorrectly attributed cutaneous leishmaniasis transmission to mosquito bites (39.9%), sandflies (64.9%), and direct contact (87.1%). In contrast, correct responses were more prevalent for transmission via air droplets (69%).

Participants demonstrated varying levels of awareness regarding the signs and symptoms of cutaneous leishmaniasis. Notably, only 22.1% recognized pruritis as a symptom, but responses were more balanced for lesions, fever, and fatigue.

Also, knowledge gaps were observed regarding the treatability (30.6% correct) and preventability (74.5% correct) of leishmaniasis. Results about leishmaniasis knowledge are presented in Table 2.

**Table 2:** Participants' response to leishmaniasis transmission questions (N=271) Leishmaniasis transmission knowledge: M±SD=6.07±1.43 Cutoff categories for the total knowledge of leishmaniasis transmission: low level n (%)=105 (38.7), moderate level n (%)=120 (44.3), and high level (%)=46 (17) n: sample size; M: mean; SD: standard deviation

Number	Description	Wrong n (%)	Correct n (%)
1	How is cutaneous leishmaniasis transmitted? (Mosquito bite)	108 (39.9)	163 (60.1)
2	How is cutaneous leishmaniasis transmitted? (Sandfly)	176 (64.9)	95 (35.1)
3	How is cutaneous leishmaniasis transmitted? (Air droplet)	84 (31)	187(69)
4	How is cutaneous leishmaniasis transmitted? (Direct contact)	236 (87.1)	35 (12.9)
5	What are the signs and symptoms of cutaneous leishmaniasis? (Pruritis)	211 (77.9)	60 (22.1)
6	What are the signs and symptoms of cutaneous leishmaniasis? (Lesion)	151 (55.7)	120 (44.3)
7	What are the signs and symptoms of cutaneous leishmaniasis? (Fever)	114 (42.1)	157 (57.9)
8	What are the signs and symptoms of cutaneous leishmaniasis? (Fatigue)	117 (43.2)	154 (56.8)
9	Which parts of the body are mostly affected? (Face)	155 (57.2)	116 (42.8)
10	Which parts of the body are mostly affected? (Arm)	140 (51.7)	131 (48.3)
11	Which parts of the body are mostly affected? (Leg)	139 (51.3)	132 (48.7)
12	Which parts of the body are mostly affected? (Two specific sites)	153 (56.5)	118 (43.5)
13	Leishmaniasis can be treated?	188 (69.4)	83 (30.6)
14	Leishmaniasis can be prevented?	69 (25.5)	202 (74.5)

Hepatitis B

The participants answered 21 questions that examined their knowledge of hepatitis B.

It is noted from the table that the mean knowledge score was 10.46±1.67. Cutoff categories further categorize the overall knowledge, with a majority falling into the "low level" (47.6%) and a smaller percentage into the "high level" (9.6%).

The majority of participants correctly identified the virus as the causative agent of hepatitis B, showcasing a commendable understanding (69.7%). While a noticeable portion correctly identified infancy as the ideal age for hepatitis B vaccination, a notable proportion provided incorrect responses, indicating gaps in awareness regarding the recommended age groups for vaccination. Results about hepatitis B knowledge are presented in Table 3.

**Table 3:** Participants' response to hepatitis B questions (N=271) Hepatitis B knowledge: M±SD=10.46±1.67 Cutoff categories for the total knowledge of hepatitis B: low level n (%)=129 (47.6), moderate level (%)=116 (42.8), and high level (%)=26 (9.6) n: sample size; M: mean; SD: standard deviation

Number	Description	Wrong n (%)	Correct n (%)
1	What is the causative agent of hepatitis B? (Virus)	82 (30.3)	189 (69.7)
2	What is the causative agent of hepatitis B? (Bacteria)	15 (5.5)	256 (94.5)
3	What is the causative agent of hepatitis B? (Protozoa)	248 (91.5)	23 (8.5)
4	What is the mode of spread of hepatitis B? (Blood transfusion)	252 (93)	19 (7)
5	What is the mode of spread of hepatitis B? (Sexual intercourse)	35 (12.9)	236 (87.1)
6	What is the mode of spread of hepatitis B? (Food)	111 (41)	160 (59)
7	What is the mode of spread of hepatitis B? (Physical contact with a person with hepatitis B)	206 (76)	65 (24)
8	What is the mode of spread of hepatitis B? (Saliva)	178 (65.7)	93 (34.3)
9	What are the signs and symptoms of hepatitis B? (Fever)	119 (43.9)	152 (56.1)
10	What are the signs and symptoms of hepatitis B? (Loss of appetite)	71 (26.2)	200 (73.8)
11	What are the signs and symptoms of hepatitis B? (Nausea and vomiting)	103 (38)	168 (62)
12	What are the signs and symptoms of hepatitis B? (Jaundice)	68 (25.1)	203 (74.9)
13	What are the measures to prevent hepatitis B? (Avoiding contaminated water)	51 (18.8)	220 (81.2)
14	What are the measures to prevent hepatitis B? (Proper cooking of food)	169 (62.4)	102 (37.6)
15	What are the measures to prevent hepatitis B? (Vaccination)	200 (73.8)	71 (26.2)
16	What are the measures to prevent hepatitis B? (Use of sterile needles and syringes)	28 (10.3)	243 (89.7)
17	What are the measures to prevent hepatitis B? (Screening blood donors)	50 (18.5)	221 (81.5)
18	Are most cases of chronic hepatitis B infection symptomatic?	52 (19.2)	219 (80.8)
19	What is the ideal age of vaccination for hepatitis B? (Infancy)	160 (59)	111 (41)
20	What is the ideal age of vaccination for hepatitis B? (Youth)	78 (28.8)	193 (71.2)
21	What is the ideal age of vaccination for hepatitis B? (Adulthood)	0 (0)	271 (100)

TB disease

Table 4 provides a comprehensive insight into participants' responses regarding TB.

**Table 4:** Participants' response to TB disease questions (N=271) TB disease knowledge: M±SD=7.007±1.90 Cutoff categories for the total knowledge of TB disease: low level n (%)=130 (48), moderate level (%)=90 (33.2), and high level (%)=51 (18.8) TB: tuberculosis; HIV: human immunodeficiency virus; n: sample size; M: mean; SD: standard deviation

Number	Description	Wrong n (%)	Correct n (%)
1	What is the cause of TB? (Virus)	169 (62.4)	102 (37.6)
2	What is the cause of TB? (Bacteria)	219 (80.9)	52 (19.5)
3	What is the cause of TB? (Fungus)	57 (21)	214 (79)
4	What are the symptoms of TB? (Cough more than two weeks)	245 (90.4)	26 (9.6)
5	What are the symptoms of TB? (Fever)	38 (14)	233 (86)
6	What are the symptoms of TB? (Weight gain)	47 (17.3)	244 (82.7)
7	What are the symptoms of TB? (Loss of appetite)	235 (86.7)	36 (13.3)
8	What are the symptoms of TB? (Abdominal pain)	104 (38.4)	167 (61.6)
9	What are the symptoms of TB? (Bloody sputum)	184 (67.9)	87 (32.1)
10	What is the route of transmission for TB? (Airborne)	52 (19.2)	219 (80.8)
11	What is the route of transmission for TB? (Waterborne)	58 (21.4)	213 (78.6)
12	What is the route of transmission for TB? (Vector-borne)	207 (76.4)	64 (23.6)
13	Which of the following can be used in the diagnosis of pulmonary TB? (Blood test)	205 (75.6)	66 (24.4)
14	Which of the following can be used in the diagnosis of pulmonary TB? (Sputum test)	169 (62.4)	102 (37.6)
15	Which of the following can be used in the diagnosis of pulmonary TB? (Urine test)	104 (38.4)	167 (61.6)
16	Which of the following can be used in the diagnosis of pulmonary TB? (Chest X-ray)	222 (81.9)	49 (18.1)
17	Are HIV patients more susceptible to getting infected with TB?	82 (30.3)	189 (69.7)
18	Is TB only transmitted by patients with active TB?	67 (24.7)	204 (75.3)

It is noted from the table that the mean knowledge score was 7.007±1.90, and to understand the mean level of participants, cutoff categories further categorize the overall knowledge, with the majority falling into the "low level" (48%) and the minimal percentage falling into the "high level" (18.8%).

Considering knowledge about TB among medical students, our findings highlight both areas of strong understanding and potential knowledge gaps, and while a substantial number correctly identified bacteria as the cause of TB, a noteworthy proportion attributed it to viruses and fungi.

Measles disease

Table 5 provides an in-depth overview of participants' responses to questions related to measles, offering insights into their understanding of the cause, symptoms, complications, transmission, and treatment of the disease.

**Table 5:** Participants' response to measles questions (N=271) Measles disease knowledge: M±SD=9.56±1.92 Cutoff categories for the total knowledge of measles disease: low level n (%)=143 (52.8), moderate level (%)=88 (32.5), and high level (%)=40 (14.8) n: sample size; M: mean; SD: standard deviation

Number	Description	Wrong n (%)	Correct n (%)
1	What is the cause of measles? (Virus)	179 (66.1)	92 (33.9)
2	What is the cause of measles? (Bacteria)	54 (19.9)	217 (80.1)
3	What is the cause of measles? (Protozoa)	237 (87.5)	34 (12.5)
4	What are the symptoms of measles? (Rush)	240 (88.6)	31 (11.4)
5	What are the symptoms of measles? (Fever)	50 (18.5)	221 (81.5)
6	What are the symptoms of measles? (Neck stiffness)	53 (19.6)	218 (80.4)
7	What are the symptoms of measles? (Chest pain)	170 (62.7)	101 (37.3)
8	What are the symptoms of measles? (Cough)	191 (70.5)	80 (29.5)
9	What are the symptoms of measles? (Conjunctivitis)	134 (49.4)	137 (50.6)
10	What are the complications of measles? (Encephalitis)	140 (51.7)	131 (48.3)
11	What are the complications of measles? (Seizures)	110 (40.6)	161 (59.4)
12	What are the complications of measles? (Flaccid paralysis)	156 (57.6)	115 (42.4)
13	What are the complications of measles? (Blindness)	192 (70.8)	79 (29.2)
14	What are the complications of measles? (Nephropathy)	188 (69.4)	83 (30.6)
15	What are the complications of measles? (Pneumonia)	187 (69)	84 (31)
16	What is the route of transmission for measles? (Airborne)	140 (51.7)	131 (48.3)
17	What is the route of transmission for measles? (Waterborne)	171 (63.1)	100 (36.9)
18	What is the route of transmission for measles? (Vector-borne)	215 (79.3)	56 (20.7)
19	Measles can be treated by antibiotics?	191 (70.5)	80 (29.5)
20	Measles is spread all over the world?	190 (70.1)	81 (29.9)

Participants exhibited a commendable understanding of measles symptoms, correctly identifying rash, fever, and neck stiffness. However, some misconceptions were noted, particularly with symptoms like chest pain, cough, and conjunctivitis. There was reasonable awareness of serious complications like encephalitis and blindness, but misconceptions were noted for flaccid paralysis and nephropathy.

Participants displayed varying levels of awareness regarding the transmission routes of measles. While many correctly identified airborne transmission, there were misconceptions related to waterborne and vector-borne transmission.

In general, it is noted from the table that the mean knowledge score was 9.56±1.92, and to understand the level of knowledge of the participants, cutoff categories further categorize the overall knowledge, with the majority falling into the "low level" (52.8%) and a minimal percentage into the "high level" (14.8%).

Cholera disease

Table 6 provides a comprehensive overview of participants' responses to questions related to cholera, offering valuable insights into their understanding of the disease's causative agent, symptoms, transmission routes, causes, and preventive measures.

**Table 6:** Participants' responses to cholera questions (N=271) Cholera disease knowledge: M±SD=14.50±1.98 Cutoff categories for the total knowledge of cholera disease: low level n (%)=135 (49.8), moderate level (%)= 100 (36.9), and high level (%)=36 (13.3) n: sample size; M: mean; SD: standard deviation

Number	Description	Wrong n (%)	Correct n (%)
1	What is the microorganism responsible for cholera? (Virus)	118 (43.5)	153 (56.5)
2	What is the microorganism responsible for cholera? (Bacteria)	216 (79.7)	55 (20.3)
3	What is the microorganism responsible for cholera? (Parasite)	108 (39.9)	163 (60.1)
4	What is the microorganism responsible for cholera? (Fungus)	216 (79.7)	55 (20.3)
5	What are the symptoms of cholera? (Bloody diarrhea)	245(90.4)	26 (9.6)
6	What are the symptoms of cholera? (Watery diarrhea)	153 (56.5)	118 (43.5)
7	What are the symptoms of cholera? (Vomiting)	54 (19.9)	217 (80.1)
8	What are the symptoms of cholera? (Shortness of breath)	72 (26.6)	199 (73.4)
9	What are the symptoms of cholera? (Joint pain)	210 (77.5)	61 (22.5)
10	What are the symptoms of cholera? (Fever)	181 (66.8)	90 (33.2)
11	What is the route of transmission for cholera? (Airborne)	87 (32.1)	184 (67.9)
12	What is the route of transmission for cholera? (Waterborne)	206 (76)	65 (24)
13	What is the route of transmission for cholera? (Vector-borne)	78 (28.8)	193 (71.2)
14	What is the route of transmission for cholera? (Feco-oral)	207 (76.4)	64 (23.6)
15	What is the route of transmission for cholera? (Direct contact)	108 (39.9)	163 (60.1)
16	What are the causes of cholera? (Poor hygiene)	195 (72)	76 (28)
17	What are the causes of cholera? (Drinking contaminated water)	73 (26.9)	198 (73.1)
18	What are the causes of cholera? (Eating contaminated food)	52 (20.3)	216 (79.7)
19	What are the causes of cholera? (Open defecation)	88 (32.5)	183 (67.5)
20	What are the causes of cholera? (Blood transfusion)	137 (50.6)	134 (49.4)
21	What are the causes of cholera? (Using contaminated needles)	191 (70.5)	80 (29.5)
22	What are the causes of cholera? (Insects)	184 (67.9)	87 (32.1)
23	What are the ways of preventing cholera? (Wash hands)	191 (70.5)	80 (29.5)
24	What are the ways of preventing cholera? (Boil water)	43 (15.9)	228 (84.1)
25	What are the ways of preventing cholera? (Kill mosquitoes)	51 (18.8)	220 (81.2)
26	What are the ways of preventing cholera? (Avoid sharing shaving blades)	167 (61.6)	104 (38.4)
27	What are the ways of preventing cholera? (Avoid sharing toilets)	146 (53.9)	125 (46.1)
28	What are the ways of preventing cholera? (Food safety)	100 (36.9)	171 (63.1)
29	What are the ways of preventing cholera? (Can't prevent)	74 (27.3)	197 (72.7)

Participants showed a strong awareness of cholera symptoms, particularly watery diarrhea and vomiting. However, misconceptions were noted with symptoms like joint pain, shortness of breath, and fever.

Participants demonstrated a good understanding of the causes of cholera, with correct identification of poor hygiene, drinking contaminated water, eating contaminated food, open defecation, and using contaminated needles. However, there was confusion about the role of blood transfusion and insects.

Also, the participants exhibited reasonable knowledge of preventive measures for cholera, including washing hands, boiling water, killing mosquitoes, and avoiding sharing toilets. However, there were misconceptions about preventing cholera through the avoidance of sharing shaving blades and the perception that cholera cannot be prevented.

It is noted from the table that the mean knowledge score was 14.50±1.98, and to understand the mean level of participants, cutoff categories further categorize the overall knowledge, with the majority falling into the "low level" (49.8%) and a minimal percentage into the "high level" (13.3%).

## Discussion

The knowledge of leishmaniasis among medical students is an important area of study. In our study, students demonstrated varying levels of awareness regarding the signs and symptoms of cutaneous leishmaniasis. The overall mean score for knowledge about leishmaniasis was 6.07, which was considered low. One study in Portugal showed that 75% of the participants reported having heard of both human and animal leishmaniasis and more than 80% reported hearing about the disease during their coursework; however, the study included both medical students and healthcare workers [21]. In the general population, knowledge about leishmaniasis varies depending on different factors, including sociodemographic and socioeconomic status. For example, a study in Yemen found that the majority of participants had basic knowledge about the curability of cutaneous leishmaniasis but held misconceptions about treatment [22].

Although medical curricula vary among different medical schools, medical students must have encountered the topic of leishmaniasis in their basic medical years, including microbiology courses, for instance. However, leishmaniasis is a neglected disease that might not have drawn the attention of medical students.

Regarding knowledge about hepatitis B, the mean score of knowledge was 10.46±1. 67, regarded as low. Although studies among medical students showed that 84.8% were aware of hepatitis B infection, the majority were unaware of the modes of transmission [23]. The latter result is comparable to ours, as 76% of medical students in our study were unaware of the mode of transition of the virus. However, another study conducted in Saudi Arabia showed a high level of knowledge among medical students regarding the modes of transmission of hepatitis B [24]. Another study conducted among medical students in Ethiopia showed that students had low awareness of the transmission modes of the virus. Also, 95.3% of the students were not vaccinated against hepatitis B, which makes them more susceptible to the infection [25]. One possible explanation for the low awareness among the students in our study is the lack of exposure to real-life cases of hepatitis B. Besides, there should be more emphasis on the disease in the exams and courses.

Considering knowledge about TB among medical students, our findings highlight both areas of strong understanding and potential knowledge gaps. While a substantial number correctly identified bacteria as the cause of TB, a noteworthy proportion attributed it to viruses and fungi. An overall mean of 7.007 is also considered low. Compared to other similar studies, Montagna et al. [26] reported that 95% of the students answered questions about TB etiology correctly; however, only 60% of the students gave the correct responses regarding clinical aspects and vaccine details. Behnaz et al. (2014) also reported a knowledge mean of 16.13±2.06 which is higher than our results. A study in China among medical students demonstrated poor knowledge about TB, with only 44.4% correct answers [27]. Another study also showed that students who encountered real TB cases or a chest X-ray for TB had more knowledge compared to the others [28]. The TB topic might not be adequately represented to students in the first years of medical sciences, TB should be presented as a public health issue, and awareness campaigns should be conducted.

Our results showed that the mean knowledge about measles among medical students at Al-Balqa Applied University was 9.56±1.92 which is also considered low. Around 79.3% of the students had no knowledge about the route of transmission, and only 33.9% knew that it is a viral disease. Generally speaking, the studies concerning knowledge about measles are few. One study showed that participants were able to answer 1.66 of these five questions about measles correctly [29]. A study among medical students in Albania showed that students at the beginning of their studies did not consider measles as a serious disease and had misunderstandings and hesitancy about the safety and efficacy of the measles vaccine [19]. The success of vaccination programs in Jordan has limited the spread of measles, and medical students are less likely to encounter cases in practice; this could be a possible explanation for the low level of knowledge among them.

The mean score for knowledge about cholera in our study was 9.56 and is considered low. Compared to previous studies, a study revealed that 35.2% of health workers had good knowledge of cholera [30]. In Lebanon, 32.5% of the general population had good knowledge about cholera [20]. The low level of knowledge among medical students could be explained by regional variations in prevalence, as cholera is somehow limited to poor areas with limited access to clean water. Another reason could be that recently much of the focus has been on new emerging diseases and chronic conditions, while cholera would not receive much attention as part of medical courses.

Strength of the study

The strength of this study lies in being one of the very few studies to address the knowledge about several communicable diseases together and, to our knowledge, the first in Jordan to address this issue.

Limitations of the study

Convenience sampling was used in this study on medical students' knowledge of communicable diseases at Al-Balqa Applied University, which may have introduced bias into the sample and limited the generalizability of the results to the larger student body. Concerns regarding recall bias and social desirability bias are also raised by the questionnaire's reliance on self-reported data, which could have an effect on the accuracy and dependability of the findings. Additionally, the study's cross-sectional approach ignores possible changes or trends in knowledge levels over time, giving only a snapshot of knowledge at a particular moment in time.

Recommendations

Our results about knowledge should be taken into consideration when reviewing the medical curricula of medical students, increasing the focus on this issue. More awareness campaigns should be conducted among medical students in order to address the problem of low knowledge. Regular training, the use of interactive learning methods, and collaboration with public health institutions are all measures to be taken into consideration to address the issue of knowledge about communicable diseases.

Future research should focus on understanding the contributing factors and understanding the relation between the different variables affecting the knowledge about communicable diseases.

## Conclusions

Our results showed a low level of knowledge among medical students regarding communicable diseases. Leishmaniasis knowledge was generally low, with notable misconceptions regarding transmission modes, symptoms, and preventability. Hepatitis B knowledge also showed areas of concern, especially regarding the modes of transmission and the recommended age for vaccination. TB knowledge had gaps, particularly in identifying the cause, symptoms, and routes of transmission. Measles knowledge indicated good awareness of symptoms but revealed misconceptions about complications and transmission routes. Cholera knowledge demonstrated a strong awareness of symptoms but exhibited confusion regarding the causative agent, transmission routes, and preventive measures.
